# Home‐based sleep monitoring reveals associations between amyloid accumulation and sleep alterations in individuals with subjective and mild cognitive impairment

**DOI:** 10.1002/alz.71326

**Published:** 2026-04-08

**Authors:** Antonia Buchal, Verena Dzialas, Elena Doering, Kathrin Giehl, Gérard N. Bischof, David Elmenhorst, Thilo van Eimeren, Alexander Drzezga, Merle C. Hoenig, for the T‐POT study group

**Affiliations:** ^1^ Department of Nuclear Medicine University of Cologne Faculty of Medicine and University Hospital Cologne Cologne Germany; ^2^ Faculty of Mathematics and Natural Sciences University of Cologne Cologne Germany; ^3^ Institute for Neuroscience and Medicine II Molecular Organization of the Brain Research Centre Juelich Juelich Germany; ^4^ Department of Neurology University of Cologne Faculty of Medicine and University Hospital Cologne Cologne Germany; ^5^ German Centre for Neurodegenerative Diseases Bonn Germany

**Keywords:** ^11^C‐PiB (amyloid) PET, cognitive impairment, EEG sleep monitoring

## Abstract

**INTRODUCTION:**

Sleep disturbances have been associated with Alzheimer's disease (AD), but their relevance in preclinical stages, such as subjective cognitive decline (SCD), and their relationship with brain pathology remain unclear.

**METHODS:**

We used a portable sleep‐monitoring headband over four consecutive nights to assess sleep in 19 cognitively unimpaired (CU), 15 SCD, and 20 mild cognitive impairment (MCI) participants with available amyloid positron emission tomography (PET). Linear‐mixed‐effects models compared sleep parameters across groups, accounting for amyloid burden, age, sex, education, and recording. Regional and voxel‐wise analyses examined regional associations between sleep parameters and amyloid burden.

**RESULTS:**

MCI patients presented reduced N3 (i.e., deep sleep), while SCD individuals showed longer N1 (i.e., light sleep) duration compared to CU. Regional amyloid burden was associated with longer light and deep sleep in amyloid‐positive individuals. Higher education was linked to better sleep efficiency.

**DISCUSSION:**

Sleep changes may serve as early indicators of cognitive dysfunction and regional amyloid accumulation.

## Highlights

- Portable EEG sleep‐monitoring headbands capture subtle sleep alterations in individuals with subjective cognitive impairment (SCD) and MCI.
- MCI patients present lower deep sleep, while individuals with SCD show longer light sleep, indicating that distinct sleep characteristics may serve as an early indicator of cognitive dysfunction.
- Regional, but not global, amyloid burden is associated with changes in deep and light sleep duration.
- Higher education is associated with greater sleep efficiency, independent of cognitive impairment and underlying brain pathology.

## 1 BACKGROUND

Sleep disturbances are increasingly recognized as both a modifiable risk factor and an early clinical feature of Alzheimer's disease (AD). Individuals with reduced sleep quantity or quality show a 1.65‐fold higher risk of subsequent cognitive decline or dementia compared with those without sleep problems.^1^ A leading mechanistic account implicates impaired glymphatic clearance, supported by experimental evidence demonstrating that disrupted sleep accelerates the accumulation of AD‐related pathology.^2^, ^3^, ^4^, ^5^ Age‐ and disease‐related sleep disturbances may be further compounded by the local build‐up of aggregated proteins, such as amyloid or tau pathology, contributing to regional neurodegeneration. These bidirectional processes are thought to create a vicious cycle in which sleep disruption facilitates pathological protein deposition, which, in turn, amplifies sleep disturbances.

Characterizing these alterations requires consideration of normal sleep architecture. Sleep typically cycles between REM and non‐REM (N1–N3) stages, with N3 duration, or deep sleep, marked by prominent slow‐wave activity (SWA). In AD, pronounced reductions in SWA consistently co‐occur with elevated amyloid beta (Aβ), the core pathological hallmark of the disease.^6^, ^7^ Regional Aβ, in turn, has been linked to disrupted SWA and impaired hippocampus‐dependent memory consolidation.^7^ Beyond SWA, AD patients also show more fragmented sleep^8^ and reduced REM sleep.^9^ Importantly, these alterations emerge early, are already detectable in mild cognitive impairment (MCI) due to AD,^10^ and worsen with disease progression, in close parallel with objective cognitive decline.^11^

Because such sleep alterations may be an early sign of ongoing brain pathology, it is critical to investigate them in preclinical populations at risk for AD, such as individuals with subjective cognitive decline (SCD). SCD individuals present themselves with self‐perceived decline not captured by neuropsychological testing. It occurs in roughly 25% of older adults above the age of 60 years and is associated with an increased risk of AD progression.^12^, ^13^, ^14^ Sleep alterations in SCD have only recently been examined, and most studies rely on actigraphy and questionnaires. Indeed, current evidence links poorer self‐reported sleep with greater SCD incidence.^15^, ^16^, ^17^ Whether these alterations are directly related to underlying brain pathology remains unclear, and objective electroencephalogram (EEG)‐based sleep‐monitoring studies in SCD are lacking. However, improving our understanding of the relationship between sleep disturbances, their onset, and underlying brain pathology in potential preclinical stages of AD may enhance early detection of at‐risk populations and deepen insight into the pathophysiological mechanisms of sleep driving disease progression in AD.

To date, polysomnography (PSG) remains the gold standard for objective sleep assessment. However, its high cost, complexity, and laboratory setting, which affect regular sleep, limit its widespread use. Recently, portable EEG sleep‐monitoring headbands (SHs) (often in combination with movement sensors) have enabled multinight assessments at participants’ homes with minimal logistic effort.^18^ Using such novel devices improves ecological validity, mitigates first‐night effects, and so allows assessment of more typical sleep behavior in familiar surroundings.^19^, ^20^ Leveraging on these advances and given the current research gaps, we aimed to (1) assess differences in sleep between individuals with unimpaired cognition and subjective and objective cognitive impairment and (2) link changes in sleep architecture to global and regional amyloid pathology, for the first time using a novel sleep‐monitoring device. To do so, we monitored sleep for up to four nights using a portable EEG headband in cognitively unimpaired (CU) individuals, individuals with SCD, and those with MCI/early AD with available amyloid PET imaging. Sleep parameters, including sleep phase duration, total sleep time (TST), wake onset after sleep (WASO), and sleep efficiency, were compared across groups and related to global amyloid burden. Moreover, we assessed the influence of established demographic factors, namely, age, sex, and education. We hypothesized that SCD and MCI patients would show shorter SWA (i.e., N3 duration) than CU, with the strongest reductions in the MCI group. We further hypothesized that higher amyloid burden would be associated with differences in sleep duration between (potential) preclinical and clinical stages and that local relationships would be observable between regional amyloid and changes in sleep architecture.

## 2 METHODS

### 2.1 Participants

In this study, 54 participants from the ongoing Tau Propagation Over Time (T‐POT)^21^ study were included. The T‐POT study is funded by the German Research Foundation and aims to characterize the progression of AD pathology. All participants were recruited from the University Hospital of Cologne. The main inclusion criteria for the T‐POT study are as follows: (1) no radiotherapy in the last 10 years, (2) absence of psychiatric diseases or claustrophobia, (3) age between 50 and 80 years, (4) no MRI contraindications. The T‐POT study participants are classified into four different groups according to established criteria by the National Institute on Aging‐Alzheimer's Association^22^, ^23^, ^24^: (1) CU adults who do not suffer from measurable objective cognitive decline or SCD; (2) individuals with SCD who report self‐perceived decline, which cannot be captured by neuropsychological testing; (3) patients with MCI and; (4) patients with AD dementia. As part of the T‐POT study, all participants undergo an elaborate multimodal neuroimaging protocol at baseline and 18‐month follow‐up. A subcohort of the T‐POT participants was offered to participate in a study on sleep depending on the date of their PET scan acquisition and their willingness to participate in the substudy.

For the current study, individuals from this sleep subcohort were selected based on the following criteria: (1) at least one night with a sleep recording quality greater than 80% (based on the quality standards provided by Beacon Biosignals); (2) TST greater than 240 min as expressed by an absolute *z*‐score greater than or equal to three; (3) (11)C‐labeled Pittsburgh Compound positron emission tomography (^11^C‐PiB) positron emission tomography (PET) scan acquisition within 1 year of sleep recording. These criteria resulted in 19 CU, 15 SCD, and 20 MCI patients (see Table 1 for an overview of participants’ characteristics), out of which five CU, five SCD, and 13 MCI were classified as amyloid‐positive based on a more liberal and intermediate Centiloid (CL) cutoff of ≥10.^25^ The sleep recordings were acquired on average 5.1 months after the PET image acquisition. The study was approved by the Ethics Committee of the University of Cologne, and all procedures were performed according to the Declaration of Helsinki. All participants provided written informed consent.

**TABLE 1:** Group and sleep characteristics.

Variable	CU (*n* = 19)	SCD (*n* = 15)	MCI (*n* = 20)	Group comparison
Participant characteristics				
Age (years)	63.68 (8.69)	65.53 (9.42)	68.75 (8.96)	*H*(2,*N* = 54) = 3.03, *p* = 0.22
Sex (M/F)	6/13	5/10	10/10	*X*(2) = 1.661, *p* = 0.44
MMSE (scores)	29.47 (0.51)	29.73 (0.49)	27.60 (2.46)	*H*(2, *N* = 54) = 10.68, *p* = 0.01^*^
Education (years)	17.16 (2.43)	17.47 (2.07)	15.75 (3.57)	*H*(2, *N* = 54) = 2.83, *p* = 0.24
Global amyloid (Centiloid)	6.51 (20.41)	8.10 (20.89)	45.52 (45.50)	*H*(2, *N* = 54) = 6.47, *p* = 0.04^*^
Sleep macrostructure (duration in minutes)				
N1	31.06 (13.21)	39.00 (17.75)	36.38 (10.43)	*H*(2, *N* = 54) = 4.39, *p* = 0.11
N2	201.08 (31.32)	188.82 (40.92)	209.35 (66.81)	*H*(2, *N* = 54) = 2.18, *p* = 0.34
N3	83.46 (26.29)	70.91 (29.65)	61.15 (28.17)	*H*(2, *N* = 54) = 6.07, *p* = 0.05^*^
REM	95.20 (29.73)	103.90 (37.27)	111.18 (64.19)	*H*(2, *N* = 54) = 0.16, *p* = 0.92
Global sleep measures				
TST (min)	410.80 (49.47)	402.63 (42.72)	418.06 (61.39)	*H*(2, *N* = 54) = 0.41, *p* = 0.81
WASO (min)	32.52 (23.28)	46.19 (31.30)	49.21 (38.46)	*H*(2, *N* = 54) = 3.25, *p* = 0.20
Sleep efficiency (%)	89.31 (5.50)	86.20 (5.29)	85.13 (7.13)	*H*(2, *N* = 54) = 3.72, *p* = 0.16

*Note*: Means and standard deviations are presented for the respective variables. The mean of the sleep measurements presents the average across the individual recordings. Chi‐squared test was used to test for differences in sex. Kruska–Wallis test was used to test differences in continuous variables.

Abbreviations: MMSE, Mini‐Mental State Examination; REM, rapid eye movement; TST, total sleep time; WASO, wake after sleep onset.

* *p* < 0.05.

### 2.2 Amyloid PET imaging

A Siemens Hybrid‐Scanner 3T MR‐PET comprising a high‐resolution BrainPET and a 3T MRT MAGNETOM Trio was used to acquire all imaging data. All participants underwent dynamic scanning for 70 min to obtain ^11^C‐PiB. All scans were averaged across timeframes obtained 40 to 70 min after injection and co‐registered with the corresponding MRI image. Spatial normalization was performed for all scans using statistical parametric mapping (SPM12; https://fil.ion.ucl.ac.uk/spm). All scans were smoothed using an 8‐mm Gaussian kernel. Standardized uptake value ratios (SUVRs) were calculated for every scan with reference to everyone's whole cerebellum. Next, a global SUVR score was extracted using a global cortical mask and converted to CL for a more accurate measure of the amyloid status.^25^ This measure was used to stratify the current cohort into amyloid‐negative and amyloid‐positive subjects based on a CL cutoff of **≥**10.

**RESEARCH IN CONTEXT**

1. **Systematic review**: Sleep disturbances have been linked to AD. However, their relevance in preclinical stages, including SCD, and their association with underlying brain pathology, remain poorly understood. Moreover, current research depends primarily on structured questionnaires or polysomnography, a logistically intensive and time‐consuming gold‐standard method, both of which limit the feasibility of evaluating sleep in real‐world environments.
2. **Interpretation**: Using a portable EEG sleep headband in combination with amyloid PET imaging information, our findings indicate that distinct sleep characteristics may signal early cognitive changes across potential preclinical (SCD) and clinical stages of AD (MCI). They further suggest that regional amyloid accumulation may contribute to sleep‐related alterations.
3. **Future directions**: Sleep alterations may serve as early indicators of cognitive dysfunction and region‐specific amyloid accumulation, highlighting a potential window for early intervention. Nonetheless, more in‐depth analyses are needed to clarify the directionality and underlying mechanisms of these associations.

### 2.3 Measurement of sleep macrostructure

Sleep architecture was assessed using the SH Dreem 3s by Beacon Biosignals (https://beacon.bio/dreem‐3s), which is a wireless device that records and stores physiological sleep data throughout a full night of sleep. All recordings were performed at participants’ homes. Two sensor types embedded in the SH can measure various sleep‐related physiological signals. Five dry EEG electrodes measure cortical brain activity, achieving seven derivations (FpZ‐O1, FpZ‐O2, FpZ‐F7, F8‐F7, F7‐O1, F8‐O2, FpZ‐F8; sampled with 250 Hz with a 0.4‐ to 35‐Hz bandpass filter). The 3D accelerometer positioned over the head captures movements, body positions, and breathing frequency. Sleep physiology can then be extracted using the automatically applied machine learning algorithm provided by Beacon Biosignals.

Participants were asked to wear the SH for a minimum of three consecutive nights. At least one recording and at maximum four recordings with sufficient quality (>80%) were necessary for inclusion in the analysis. The following parameters were extracted from the individual recordings: (1) TST, (2) wake onset after sleep, (3) sleep efficiency, (4) sleep phase durations (N1, N2, N3, REM) in minutes, as well as the receptive percentages.

### 2.4 Statistical analysis

#### 2.4.1 Linear mixed model analysis

To examine the independent effects of diagnostic group status and global amyloid on extracted sleep parameters, we performed linear mixed‐effects modeling (LMM) using R Studio (package: lme). Because not all participants were amyloid positive, both diagnostic group and amyloid burden were included as fixed effects to estimate their independent contributions. The LMMs included the respective sleep parameters (i.e., sleep phase durations, TST, WASO, sleep efficiency) up to four recordings as dependent variables, adding up to a total of 174 recordings across 54 subjects (CU *n*[recording] = 62, SCD *n*[recording] = 49, MCI *n*[recording] = 63). The following continuous fixed effects were introduced: global amyloid burden (CL value), education, age, and recording number. Ordinal fixed effects comprised sex (male [M]/female [F]) and group status (CU, SCD, MCI), with the CU group being the reference. Random intercepts and slopes for the number of recordings were specified for each subject ID to account for repeated measurements. Because adaptation effects across nights (first‐night effect) were expected, random intercepts and slopes for recording number were specified for each subject ID to account for both individual baseline differences and subject‐specific adaptation trajectories across repeated measurements. Models were estimated using restricted maximum likelihood (REML). Standard model assumptions (linearity, homoscedasticity, normality of residuals, and influential observations) were evaluated. In case of severe outlier recordings, they were removed based on the normalized residual value >3 standard deviations. Significance level was set at *p* < 0.05. Given our predefined directionality of effects according to our hypotheses, the models were not corrected for multiple comparisons.

### 2.5 Regional and voxel‐based analysis of regional amyloid burden and light and deep sleep duration

#### 2.5.1 Regional analysis

As the global amyloid burden did not show a significant association with sleep measures and only a trend‐level effect for N3 duration, additional analyses focused on regional amyloid burden. Regions of interest (ROIs) were selected a priori based on previous evidence linking amyloid deposition and sleep alterations in AD and on their known vulnerability to early amyloid accumulation. These included the medial prefrontal cortex, anterior cingulate cortex, posterior cingulate cortex, and precuneus.^4^ The insula, although not typically affected in the early stages of the disease,^26^ was additionally included due to prior reports suggesting a potential relationship with sleep‐related alterations in AD.^4^

Mean SUVR values were extracted for each region using the Automated Anatomical Labeling (AAL3) atlas.^27^ Linear mixed‐effects models identical to those described above were then applied, replacing the global CL measure with regional SUVR values. To avoid collinearity between regional predictors, separate models were fitted for each region. False discovery rate (FDR) correction was applied to account for multiple comparisons. In total, 10 models were estimated, corresponding to five regions for each of the two sleep phases (N1 and N3).

#### 2.5.2 Voxel‐based analysis

Based on the LMM results, we additionally conducted a regional voxel‐wise whole‐brain analysis in the amyloid‐positive subcohort (five CU, five SCD, 13 MCI) using the spatially normalized, intensity‐standardized (reference region: whole cerebellum), and smoothed (8‐mm full width at half maximum) ^11^C‐PiB PET scans. Two separate models were run with either mean N1 duration or mean N3 duration as the independent variable, controlling for age, sex, and education. Mean sleep duration was computed as the average across all available recordings per subject. A cortical gray‐matter mask constrained regional comparisons. Given the modest sample size, voxel‐wise results were first evaluated at an uncorrected threshold of *p* < 0.001 and subsequently at more liberal thresholds of *p* < 0.005 and *p* < 0.01 to assess the robustness and spatial consistency of the observed clusters. In addition, sensitivity analyses were conducted using more conservative definitions of amyloid positivity, applying CL cutoffs of ≥20 and ≥30.

Significant clusters from this voxel‐based discovery analysis were saved as fixed masks and used to extract mean regional SUVR for all participants (amyloid‐positive and amyloid‐negative). For validation purposes, we then tested whether the sleep–amyloid associations differed by amyloid status (CL > 10) using LMM, including the interaction term of mean SUVR of either N1 and N3 and amyloid group in addition to age, sex, and education as covariates, given the results of our previous LMM. The linear mixed‐effects models again comprised a random intercept and recording slope for each participant to account for repeated recordings.

## 3 RESULTS

3.1

The three groups did not differ in terms of age, sex, or years of education but showed, as per design, expected differences in global amyloid burden (*H*[2, *N* = 54] = 6.47, *p* = 0.04) and cognitive function (—*H*[2, *N* = 54] = 10.68, *p* = 0.01).

### 3.2 Effect of group status and demographic factors on sleep parameters

**FIGURE 1:**
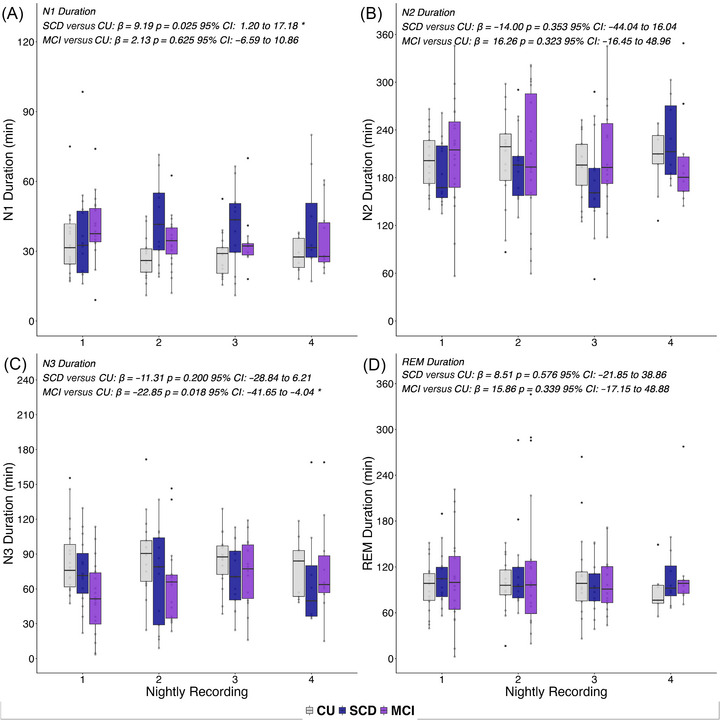
Group differences in macrostructure of sleep. Duration in minutes of each sleep phase for each group across the four nights is depicted. Groups are color‐coded, and significant differences are highlighted with an asterisk.

MCI patients showed reduced N3 duration (*ß* = −22.85; *p* = 0.02; 95% CI −41.65 to −4.04), while SCD subjects presented longer N1 duration (*ß* = 9.19; *p* = 0.03; 95% CI: 1.20 to 17.18) in comparison to the CU group. The groups did not differ in the other tested sleep parameters (i.e., N2, REM, TST, WASO, sleep efficiency). For an illustration of effects, see Figure 1.

Moreover, greater sleep efficiency was associated with higher education (*ß* = 0.76; *p* = 0.01; 95% CI: 0.16 to 1.36) and younger age (*ß* = −0.17; *p* = 0.07; 95% CI: −0.36 to 0.01). A positive effect of age was found for WASO (*ß* = 0.82; *p* = 0.01; 95% CI: 0.20 to 1.44), N1 (*ß* = 0.43; *p *= 0.03; 95% CI: 0.05 to 0.82), and N2 (*ß* = 2.14; *p* < 0.001; 95% CI: 0.70 to 3.58) duration, and a negative effect on N3 duration (*ß* = −0.95; *p* = 0.03; 95% CI −1.77 to −0.12). Lastly, females presented shorter N3 duration in comparison to males across diagnostic groups (*ß* = 18.06; *p* = 0.03; 95% CI: 2.33 to 33.79).

Key statistics for all tested models are listed in Table 2. Similar results were obtained when using the percentage values instead of sleep phase durations in minutes (for details, see Table S1 and Figure S1 in the supplementary information).

**TABLE 2:** Associations between sleep phases, clinical status, and global amyloid.

Predictor	N1 duration	N2 duration	N3 duration	REM duration	TST	WASO	Sleep efficiency
SCD versus CU	***ß*** **= 9.19, *p* = 0.03** **95% CI: [1.20, 17.18]**	*ß* = −14.00, *p* = 0.35 95% CI: [−44.04, 16.04]	*ß* = −11.31, *p* = 0.20 95% CI: [−28.84, 6.21]	*ß* = 8.51, *p* = 0.58 95% CI: [−21.85, 38.86]	*ß* = −9.86, *p* = 0.57 95% CI: [−44.13, 24.41]	*ß* = 9.50, *p* = 0.15 95% CI: [−3.61, 22.60]	*ß* = −2.57, *p* = 0.19 95% CI: [−6.43, 1.29]
MCI versus CU	*ß* = 2.13, *p* = 0.62 95% CI: [−6.59, 10.86]	*ß* = 16.26, *p* = 0.32 95% CI: [−16.45, 48.96]	***ß* = −22.85, *p* = 0.02** **95% CI: [−41.65, −4.04]**	*ß* = 15.86, *p* = 0.34 95% CI: [−17.15, 48.88]	*ß* = 6.98, *p* = 0.71 95% CI: [−30.08, 44.04]	*ß* = 8.19, *p* = 0.24 95% CI: [−5.71, 22.09]	*ß* = −1.76, *p* = 0.41 95% CI: [−5.97, 2.46]
Centiloid	*ß* = −0.02, *p* = 0.74 95% CI: [−0.13, 0.09]	*ß* = −0.40, *p* = 0.06 95% CI: [−0.80, 0.01]	*ß* = 0.24, *p* = 0.05 95% CI: [−0.00, 0.47]	*ß* = −0.03, *p* = 0.88 95% CI: [−0.44, 0.38]	*ß* = −0.12, *p* = 0.61 95% CI: [−0.58, 0.34]	*ß* = −0.07, *p* = 0.42 95% CI: [−0.24, 0.10]	*ß* = 0.01, *p* = 0.70 95% CI: [−0.04, 0.06]
Age	***ß* = 0.43, *p* = 0.03** **95% CI: [0.05, 0.82]**	***ß* = 2.14, *p* < 0.001** **95% CI: [0.70, 3.58]**	***ß* = −0.95, *p* = 0.03** **95% CI: [−1.77, −0.12]**	*ß* = −0.24, *p* = 0.75 95% CI: [−1.69, 1.22]	*ß* = 1.24, *p* = 0.14 95% CI: [−0.40, 2.89]	***ß* = 0.82, *p* = 0.01** **95% CI: [0.20, 1.44]**	***B *= −0.17, *p* = 0.07** **95% CI: [−0.36, 0.01]**
Males versus Females	*ß* = −5.58, *p* = 0.13 95% CI: [−12.77, 1.62]	*ß* = 17.07, *p* = 0.21 95% CI: [−9.99, 44.12]	***ß* = 18.06, *p* = 0.03** **95% CI: [2.33, 33.79]**	*ß* = −18.47, *p* = 0.18 95% CI: [−45.80, 8.86]	*ß* = 14.39, *p* = 0.35 95% CI: [−16.41, 45.19]	*ß* = −0.38, *p* = 0.95 95% CI: [−12.10, 11.34]	*B *= −0.01, *p *= 1.00 95% CI: [−3.48, 3.47]
Education	*ß* = −0.48, *p* = 0.44 95% CI: [−1.73, 0.77]	*B *= 0.33, *p* = 0.89 95% CI: [−4.37, 5.03]	*ß* = 1.56, *p* = 0.25 95% CI: [−1.16, 4.29]	*ß* = 0.61, *p* = 0.80 95% CI: [−4.14, 5.37]	*ß* = 1.42, *p* = 0.59 95% CI: [−3.92, 6.77]	*ß* = −0.36, *p *= 0.73 95% CI: [−2.43, 1.71]	***ß* = 0.76, *p* = 0.01** **95% CI: [0.16, 1.36]**
Recording	*ß* = −0.78, *p* = 0.37 95% CI: [−2.47, 0.92]	*ß* = 2.22, *p* = 0.49 95% CI: [−4.09, 8.53]	*B *= 1.95, *p* = 0.31 95% CI: [−1.87, 5.77]	*ß* = 0.83, *p* = 0.78 95% CI: [−5.05, 6.71]	*ß* = 4.34, *p* = 0.36 95% CI: [−5.04, 13.73]	*ß* = 0.42, *p* = 0.80 95% CI: [−2.97, 3.82]	*ß* = 0.74, *p* = 0.23 95% CI: [−0.49, 1.97]

*Note*: Results of linear mixed model analyses assessing the interplay between group status, global amyloid pathology, and demographic variables on sleep parameters (N1, N2, N3, REM, TST, WASO, and sleep efficiency) are listed. Each cell reports the unstandardized *ß* coefficient (*ß*), *p* value (*p*), and 95% CI. Significant results (*p* < 0.05) are highlighted in bold.

Abbreviations: CI, confidence interval; REM, rapid eye movement; TST, total sleep time; WASO, wake after sleep onset.

### 3.3 Effect of global and regional amyloid burden on sleep parameter

**FIGURE 2:**
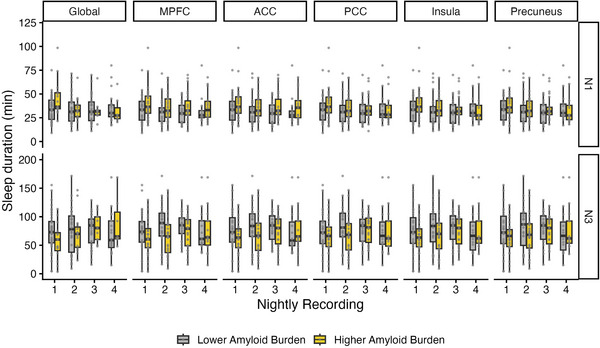
Associations between regional amyloid burden and sleep duration across nightly recordings. Boxplots show N1 and N3 sleep duration in minutes for each recording night, stratified by amyloid burden within each region of interest. For visualization purposes, global amyloid burden was defined using a Centiloid cutoff of ≥10 (Amy− vs Amy+). For regional analyses (including MPFC, ACC, PCC, insula, precuneus), participants were categorized into lower and higher amyloid burden groups using a median split of the respective regional standardized uptake value ratio values. Panels are arranged by sleep stage (rows) and region (columns). *Y*‐axis limits differ between N1 and N3 to reflect their respective duration ranges. ACC, anterior cingulate cortex; MPFC, medial prefrontal cortex; PCC, posterior cingulate cortex.

No significant effect of global amyloid burden was observed on any of the tested parameters. However, a positive trend effect was found in terms of N3 duration and global amyloid burden (*ß* = 0.24; *p* = 0.05; 95% CI: −0.00 to 0.47). The regional analysis across the five predefined ROIs did not reveal a significant association between regional amyloid burden and either N1 or N3 sleep duration across the four recording nights (Figure 2). Upon visual inspection, higher global and regional amyloid burden appeared to be associated with longer N1 and shorter N3 duration during the first night. However, this pattern was not observed consistently across the remaining nights and often reversed, possibly reflecting variability in individual recordings.

**FIGURE 3:**
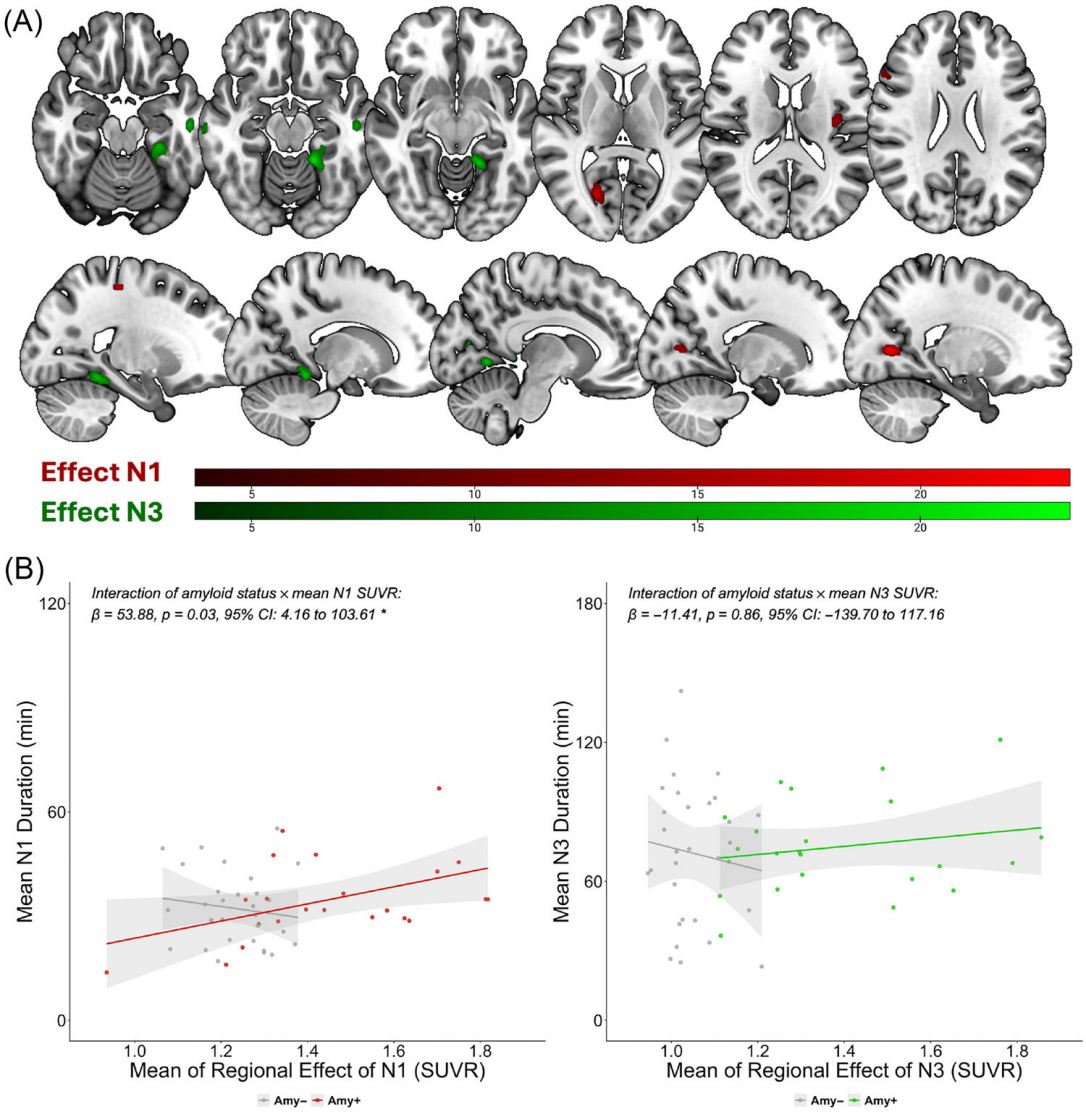
(A) In amyloid‐positive participants, clusters showing positive associations between regional amyloid burden (standardized uptake value ratio from (11)C‐labeled Pittsburgh Compound B positron emission tomography) and mean sleep duration are displayed (N1 in red; N3 in green). Maps are thresholded at an uncorrected *p* < 0.001; the color bar indicates T values. (B) Interaction between regional amyloid burden in the clusters shown in (A) and sleep stage duration depending on amyloid status (negative vs positive; Centiloid ≥ 10) in full cohort. Lines depict fitted slopes from linear mixed‐effects models across all recordings. For better clarity, points show subject‐level mean sleep values instead of the individual recordings.

The voxel‐based analysis in the amyloid‐positive subcohort yielded several clusters surpassing an extent threshold of 50 voxels that were associated with greater N1 and N3 duration. The associations between increased N1 duration and amyloid burden were observed in the calcarine sulcus, insula, inferior frontal gyrus, and postcentral gyrus. The relation between increased N3 duration and regional amyloid, in contrast, was confined to temporal regions, comprising the middle temporal gyrus, parahippocampal gyrus, fusiform gyrus, and lingual gyrus. Notably, for both N1 and N3 duration, a positive effect was found. Reverse contrasts did not yield significant clusters. Regional associations are depicted in Figure 3A. The spatial cluster patterns remained largely consistent across analyses using different CL cutoffs and voxel‐wise thresholds and were also preserved when *z*‐transformed maps based on the amyloid‐negative reference group were used (Supplementary Information, pp. 3–5).

The subsequent validation analyses, including the entire cohort of amyloid‐positive and amyloid‐negative subjects, yielded a significant interaction between amyloid status and mean ROI SUVR for N1 duration (*β* = 53.88, *p* = 0.03, 95% CI: 4.16 to 103.61) but not for N3 duration (*β* = −11.41, *p* = 0.86, 95% CI: −139.70 to 117.16). In terms of the N1 cluster, there occurred one outlier in the amyloid‐positive group with a mean SUVR below 1.0. Upon removal of the outlier from the interaction analysis, including the N1 effect, the results remained significant (*β* = 33.54, *p* = 0.04, 95% CI: 2.27 to 64.82). The results for both N1 and N3 interaction effects are visualized in Figure 3B.

## 4 DISCUSSION

In this study, we leveraged a portable EEG‐based home sleep‐monitoring device to characterize how sleep architecture relates to amyloid pathology across the AD continuum. Consistent with findings using PSG, individuals with MCI exhibited a marked reduction in N3 duration. Importantly, we were able to provide the first evidence that individuals with SCD already show measurable alterations in sleep architecture, namely a significant increase in N1 duration and a trend toward reduced N3, relative to CU individuals. This suggests that subtle sleep‐stage disruptions may emerge even before objective cognitive impairment becomes clinically detectable. Additionally, we found that sleep efficiency was positively associated with educational attainment, supporting a potential protective role of cognitive reserve against age‐ and disease‐related sleep disruption. With respect to amyloid pathology as a potential driver and facilitator of sleep disturbances, we did not find an effect of the tested sleep parameters and global amyloid burden or amyloid burden in predefined ROIs. Yet, voxel‐wise analyses revealed discrete region‐specific relationships between amyloid accumulation and altered sleep architecture, particularly within small clusters associated with the insula, inferior temporal cortex, and frontal regions.

Consistently, sleep disturbances have been linked to deficits in memory and attention in aging and disease.^28^, ^29^ The observed alterations in sleep durations may therefore contribute to both the subjective cognitive complaints reported in SCD and the overt impairments seen in the MCI group. Reductions in N3 duration have been associated not only with increased risk of dementia^6^ but also with the severity of cognitive deficits across neurodegenerative disorders, including Alzheimer's and Parkinson's disease.^30^, ^31^ Likewise, alterations in sleep macrostructure have been tied to poorer cognitive performance, particularly in memory consolidation.^32^, ^33^, ^34^ Reduced deep sleep (i.e., N3) may disrupt memory consolidation, contributing to subtle subjective cognitive complaints in SCD and to more pronounced deficits as the disease progresses to MCI due to AD. Importantly, beyond the trend toward reduced SWA (i.e., N3 duration), the SCD group also showed significantly longer N1 duration, hence light sleep, compared to CU individuals. Recent work suggests that a shift toward lighter sleep stages may represent a compensatory mechanism, which preserves sleep continuity or cognitive function in the face of early neurodegeneration.^35^ Hence, an increase in N1 duration as observed in the SCD cohort may reflect an adaptive response, helping to sustain cognitive performance. This assumption, however, requires further longitudinal assessments linking changes in sleep patterns to cognitive function over time.

While sleep disturbances are thought to contribute to cognitive dysfunction, they have also been implicated in the accumulation of brain pathology through impaired clearance of soluble protein waste.^36^ In turn, greater pathology burden is believed to exacerbate sleep disturbances, creating a vicious cycle. We therefore examined the association between global and regional amyloid burden and sleep parameters across groups. No significant relationships were observed between global amyloid burden and changes in sleep architecture. Moreover, when assessing the association between sleep parameters and amyloid burden in predefined ROIs previously implicated in sleep‐related alterations in AD,^4^ no significant effects were observed across recording nights. Nonetheless, upon visual inspection, higher regional amyloid burden was associated with longer N1 duration and shorter N3 duration during the first night. However, this pattern was not evident across subsequent nights, which may reflect night‐to‐night variability in sleep measures as well as the smaller number of amyloid‐positive participants, limiting statistical power to detect consistent effects.

Importantly, the subsequent voxel‐wise analysis revealed focal links between amyloid and both light (N1) and deep (N3) sleep within temporal and frontal cortices. This is consistent with the recent proposition that regional deposition, particularly in fronto‐cingulate and hippocampal areas, is more closely associated with sleep alterations than global burden.^4^ In the amyloid‐positive subset, longer N1 duration was associated with higher regional SUVRs in the insula, frontal, and calcarine cortex, which are regions implicated in sleep–wake regulation.^37^, ^38^, ^39^ Local amyloid aggregation in these areas may disturb cortical dynamics, promoting lighter sleep and affecting downstream cognitive effects on alertness, attention, and memory.

Unexpectedly, longer N3 duration was associated with greater local amyloid burden in temporal regions (parahippocampal and middle temporal gyri). Given that individuals with MCI exhibited overall lower N3 than controls, a negative relationship would have been anticipated. The opposite direction, together with the absence of moderation by amyloid status for N3, suggests that SWA reductions along the AD continuum may be driven by processes less tightly coupled to regional amyloid but potentially rather by tau pathology, age‐related changes, or atrophy. Because the N3 clusters were localized to temporal regions, follow‐up analyses relating SWA metrics to tau pathology are warranted. Furthermore, future investigations should more precisely characterize the spectral subcomponents of N3 sleep. Evidence suggests a dissociation wherein low‐frequency SWA relates to reduced amyloid burden^7^, ^18^, ^40^ and slower accumulation over time,^41^ whereas high‐frequency SWA is associated with greater burden^7^ and accelerated accumulation^41^.

Beyond amyloid burden and group status, we also examined the influence of education, age, and sex. Higher education was associated with better sleep efficiency, independent of pathology burden and consistent with prior work in CU adults.^42^ This finding supports the notion that education may confer both resistance to pathology accumulation and resilience against its cognitive effects.^43^ Further studies should investigate how lifetime and lifestyle factors interact with sleep, as this may inform preventive strategies for AD.

As expected, older age was linked to shifts toward lighter sleep, reduced deep sleep, and lower sleep efficiency.^44^, ^45^, ^46^ Sex differences were also observed. While women are typically reported to spend more time in deep sleep than men,^47^ our sample showed the opposite pattern, with women spending less time in N3. This discrepancy may be partly due to the unbalanced sex distribution in our cohort, limiting the generalizability of these findings.

Several limitations should be considered when interpreting these findings. First, the relatively small sample size warrants replication in larger cohorts with a more balanced sex distribution to enhance statistical power and the reliability of effect estimates. In addition, the asymmetry of the observed associations, including effects in brain regions such as the insula, not typically implicated in early amyloid pathology, suggests that larger samples are needed to clarify the generalizability and biological significance of these findings. Moreover, some clusters were located in proximity to white matter regions. Although analyses were restricted to gray matter using gray matter masking procedures, and the spatial pattern was replicated using *z*‐score maps (based on the amyloid‐negative reference sample), a residual influence of white matter signal cannot be entirely excluded. Aside from this, further investigations on the regional associations of AD pathology, in particular tau, and individual changes in sleep are warranted. In this regard, longitudinal designs will shed light on the association between sleep disturbances and the build‐up of AD pathology across the AD continuum.

Despite its preliminary nature, this is, to our knowledge, the first study using a novel wearable sleep‐monitoring device in combination with amyloid PET imaging in subjects with SCD and MCI. Our data suggest that distinct sleep characteristics may serve as an early indicator of cognitive dysfunction in SCD and MCI populations. Cognitive impairment potentially due to stage‐dependent sleep characteristics may, in turn, be driven by the regional accumulation of amyloid burden. A clearer understanding of how sleep alterations contribute to cognitive decline and regional pathology accumulation in AD will be critical for elucidating the neuromodulatory role of sleep. Ultimately, such insights may inform the development of sleep‐based interventions as preventive or therapeutic strategies in populations at risk for AD.

## CONFLICT OF INTEREST STATEMENT

A.B., V.D., E.D., and D.E. report no conflicts of interest related to this work. K.G. received speaker honoraria from Synaptikon GmbH/NeuroNation MED. G.N.B. is a full‐time employee of Life Molecular Imaging (LMI), a Lantheus Company. T.v.E. reports consulting fees from Lundbeck Foundation and Gain Therapeutics; payment for lectures from Eisai Germany; participation on a data safety monitoring board or advisory board from ICON; stock or stock options in NVIDIA, IBM, and Microsoft. A.D. reports research support from Siemens Healthineers, Life Molecular Imaging, GE Healthcare, AVID Radiopharmaceuticals, Sofie, Eisai, Novartis/AAA, and Ariceum Therapeutics and speaker honorary/advisory board commitments for Siemens Healthineers, Sanofi, GE Healthcare, Biogen, Novo Nordisk, Invicro, Novartis/AAA, Bayer Vital, Lilly, Peer View Institute for Medical Education, International Atomic Energy Agency, and Swiss Rockets. He holds stocks in Siemens Healthineers, Lantheus Holding, and Lilly. He participates in trials, including principal investigator roles in industry‐sponsored trials, e.g., by Novartis Pharma, and holds a patent for 18F‐JK‐PSMA‐7 (PSMA PET imaging tracer). 2‐Alkoxy‐6‐[18F]Fluoronicotinoyl substituted Lys‐C(O)‐Glu derivatives as efficient probes for imaging of PSMA expressing tissues (Patent No.: EP3765097A1; Date of patent: January 20, 2021). M.C.H. reports the provision of 15 sleep‐monitoring headbands for research purposes by the Dreem company (now Beacon Biosignals) as part of a research proposal prize.

## CONSENT

All participants provided written informed consent prior to inclusion in the study.

## Supporting information



Supporting Information

Supporting Information
