# Supplementary material for: Home‐based sleep monitoring reveals associations between amyloid accumulation and sleep alterations in individuals with subjective and mild cognitive impairment
Source: Alzheimers Dement. 2026 Apr 8;22(4):e71326. doi: 10.1002/alz.71326 (PMC13060651; doi:10.1002/alz.71326)
Supplement: Supplementary file 1 — Supporting Information [file ALZ-22-e71326-s001.docx]

**Results based on percentage values of each sleep phase:**

Percentage values were computed relative to the total sleep time of each individual. The LMM yielded similar effects as the LMM based on the duration in minutes per sleep phase.

| **Predictor** | **N1 Percentage** | **N2 Percentage** | **N3 Percentage** | **REM Percentage** |
| --- | --- | --- | --- | --- |
| **SCD vs. CU** | **B=2.16, p=0.03 CI95%: [0.27, 4.05]** | B=-2.74, p=0.37 CI95%: [-8.79, 3.31] | B=-2.27, p=0.32 CI95%: [-6.78, 2.23] | B=2.69, p=0.37 CI95%: [-3.26, 8.64] |
| **MCI vs. CU** | B=0.23, p=0.82 CI95%: [-1.82, 2.28] | B=0.80, p=0.81 CI95%: [-5.85, 7.46] | **B=-5.42, p=0.03 CI95%: [-10.27, -0.56]** | B=2.88, p=0.38 CI95%: [-3.67, 9.42] |
| **Centiloid** | B=0.00, p=0.83 CI95%: [-0.02, 0.03] | B=-0.07, p=0.08 CI95%: [-0.16, 0.01] | B=0.06, p=0.07 CI95%: [-0.00, 0.12] | B=0.01, p=0.75 CI95%: [-0.07, 0.09] |
| **Age** | B=0.07, p=0.10 CI95%: [-0.02, 0.16] | **B=0.37, p=0.02 CI95%: [0.07, 0.66]** | **B=-0.26, p=0.02 CI95%: [-0.47, -0.04]** | B=-0.21, p=0.15 CI95%: [-0.50, 0.08] |
| **Females vs. Males** | B=-1.33, p=0.12 CI95%: [-3.03, 0.37] | B=2.28, p=0.40 CI95%: [-3.18, 7.74] | B=3.64, p=0.08 CI95%: [-0.41, 7.69] | B=-4.56, p=0.09 CI95%: [-9.93, 0.81] |
| **Education** | B=-0.14, p=0.35 CI95%: [-0.43, 0.16] | B=-0.04, p=0.93 CI95%: [-1.00, 0.91] | B=0.28, p=0.42 CI95%: [-0.42, 0.98] | B=0.03, p=0.94 CI95%: [-0.90, 0.97] |
| **Recording** | B=-0.28, p=0.13 CI95%: [-0.64, 0.08] | B=-0.06, p=0.92 CI95%: [-1.11, 1.00] | B=0.34, p=0.42 CI95%: [-0.51, 1.19] | B=-0.09, p=0.87 CI95%: [-1.19, 1.00] |

*S-Table 1 – Results of linear mixed model analyses assessing the effect of group status, global amyloid pathology and demographic variables on the percentage of each sleep phase duration relative to the total sleep time. Each cell reports the unstandardized ß-coefficient (B), p-value (p) and 95% confidence interval (CI). Significant results (p < .05) are highlighted in bold. REM= rapid-eye movement.*

*
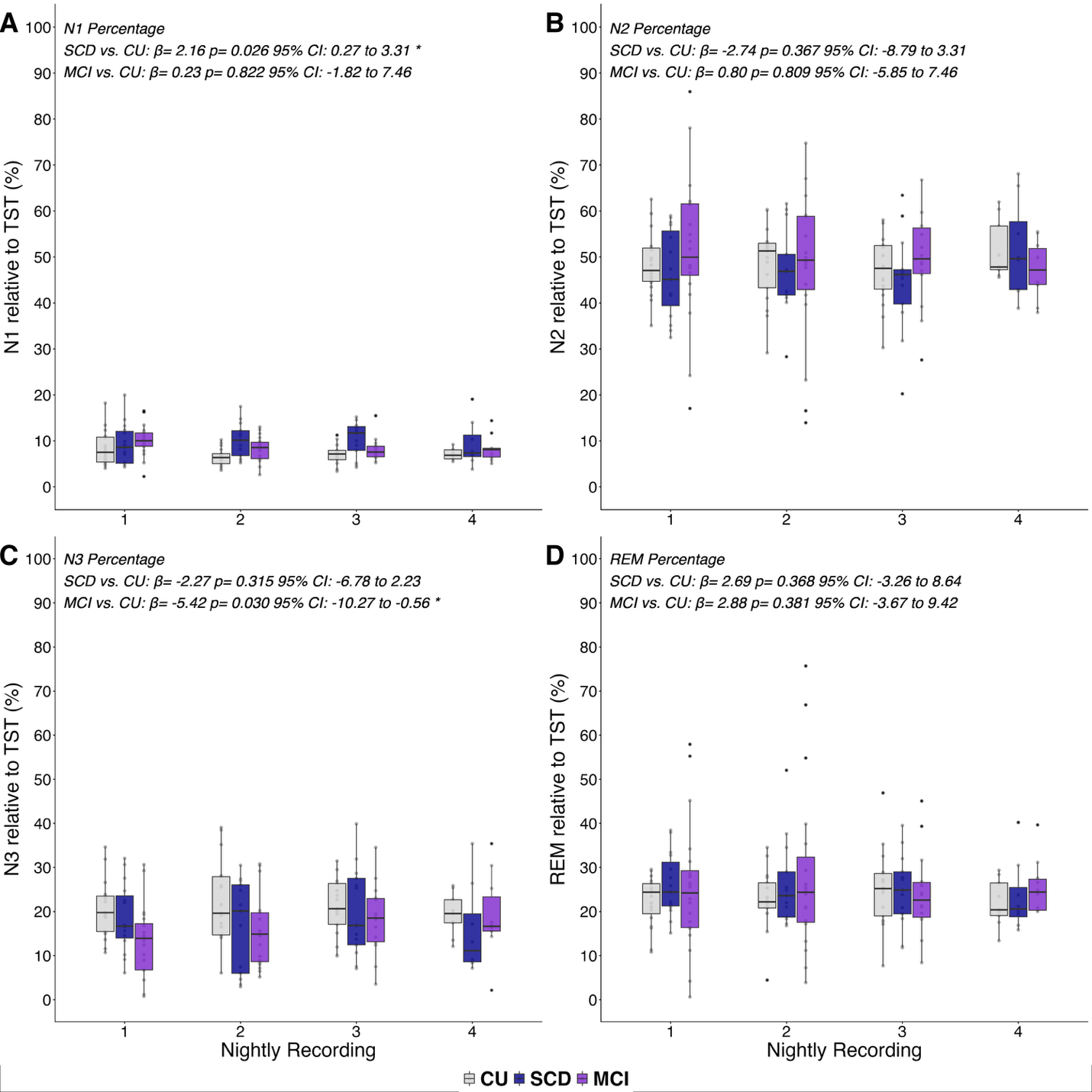
*

*S- Figure 1 – Group differences in the macrostructure of sleep. Percentage values of each sleep phase relative to the total sleep time is depicted for each group across the four nights. Groups are color-coded and significant differences are highlighted with an asterisk. TST = Total sleep time.*

**Voxel-wise associations between N1 and N3 duration using different Centiloid thresholds for amyloid positivity classification and p-value thresholds:**

To evaluate the robustness of the findings with respect to the definition of amyloid positivity, all voxel-wise analyses were repeated using more conservative Centiloid cut-offs of 20 CL and 30 CL. Using a threshold of ≥ 30 CL, 13 participants were classified as amyloid-positive, whereas a cut-off of ≥ 20 CL resulted in 18 amyloid-positive individuals. Owing to the reduced number of amyloid-positive participants at these stricter Centiloid thresholds, voxel-wise statistical evaluation was performed at a threshold of p < .005 (uncorrected). The resulting cluster distributions for these sensitivity analyses are provided below. Given the limited degrees of freedom in the ≥ 30 CL subgroup (df = 8), these results should be interpreted with caution.

| **N1 Positive Contrast for p<.005** | | |
| --- | --- | --- |
| CL10 (n=23) | CL20 (n=18) | CL30 (n=13) |
| 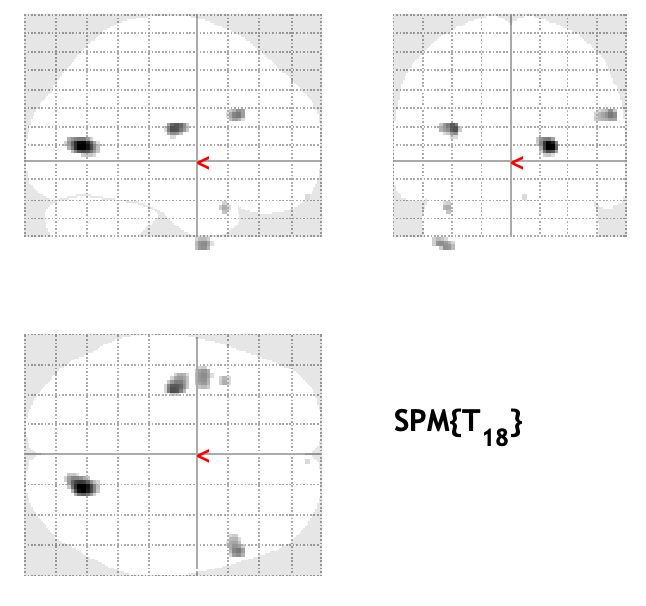 | 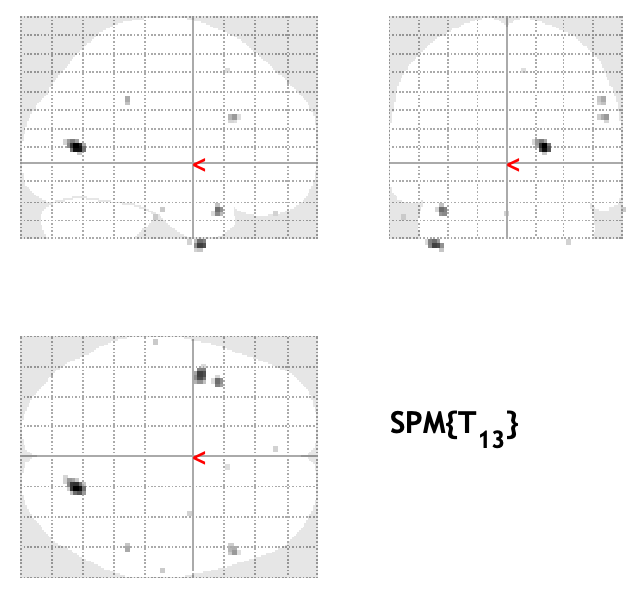 | 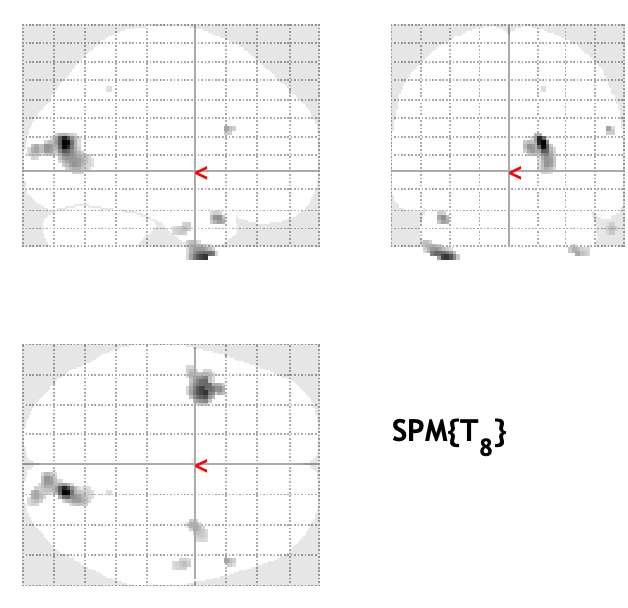 |
| **N3 Positive Contrast for p<.005** | | |
| CL10 (n=23) | CL20 (n=18) | CL30 (n=13) |
| 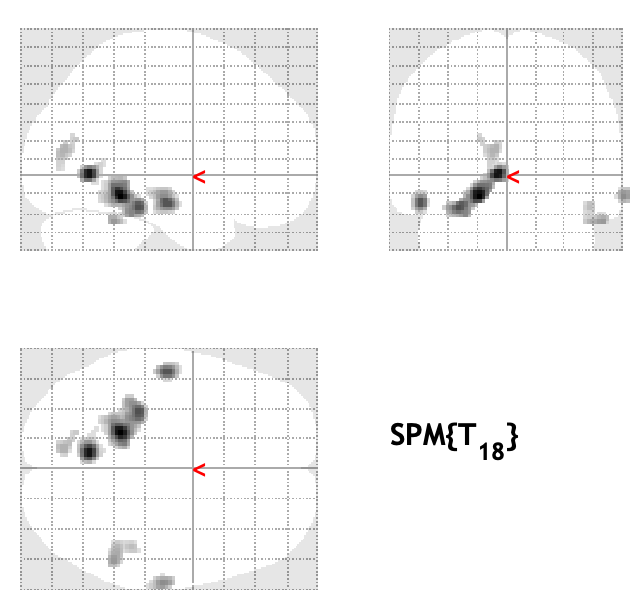 | 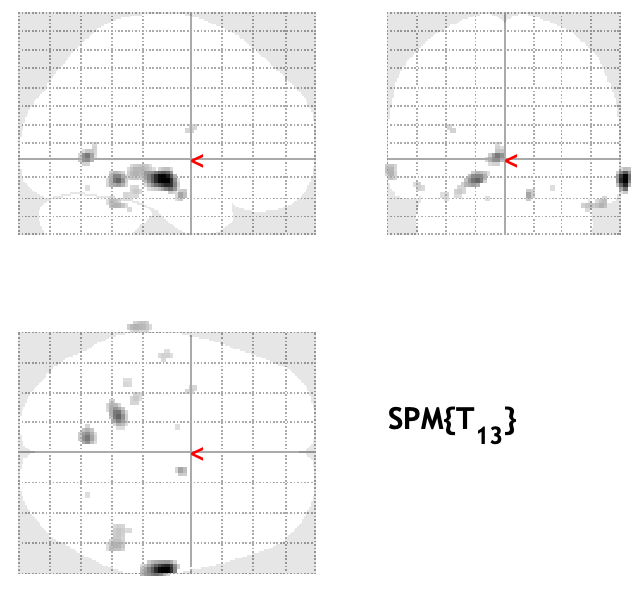 | 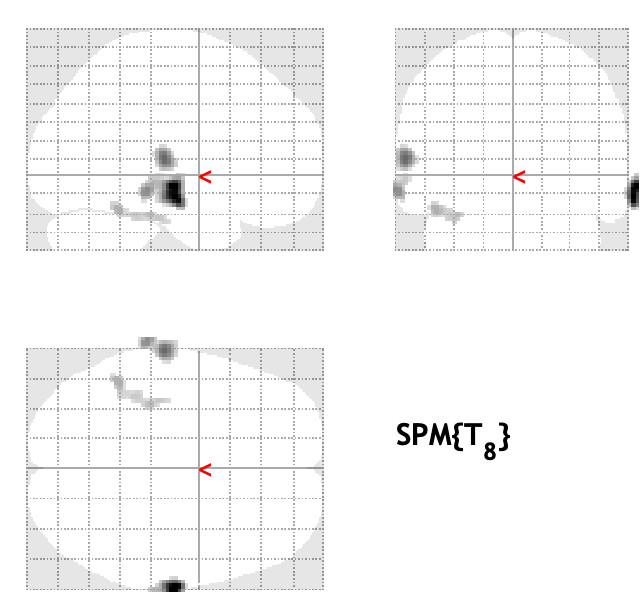 |

Additionally, we assessed the spatial cluster pattern at different p-thresholds in the amyloid positive group based on a CL cut-off of ≥ 10CL. With lowering the p-threshold, the clusters became more symmetric.

| **Positive Effect of N1 in CL10 group** | | | |
| --- | --- | --- | --- |
| P < .001  T = 3.61 | P < .005  T = 2.88 | P < .01  T = 2.55 | P < .05  T = 1.73 |
| 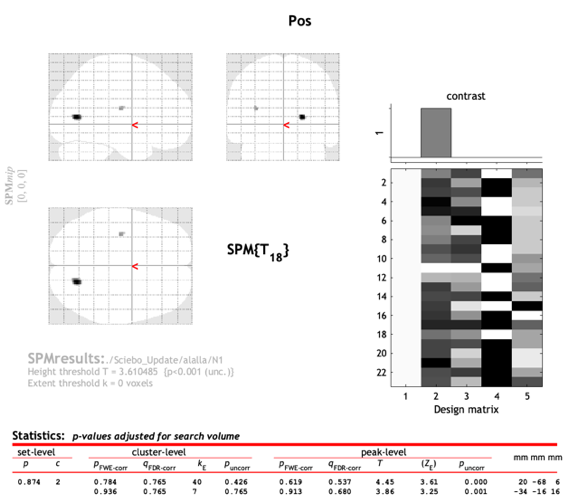 | 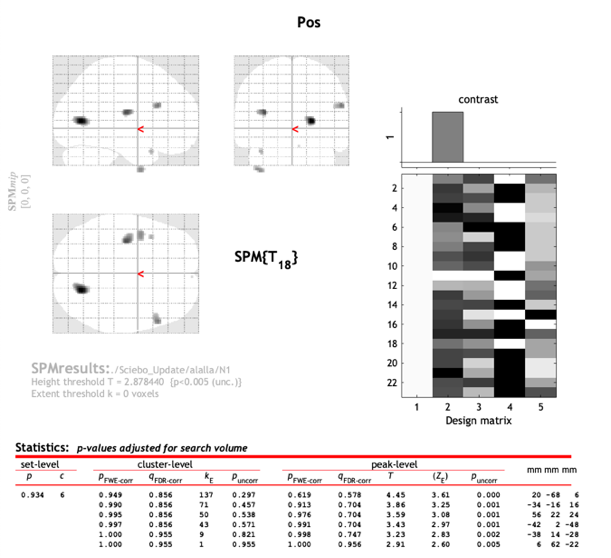 | 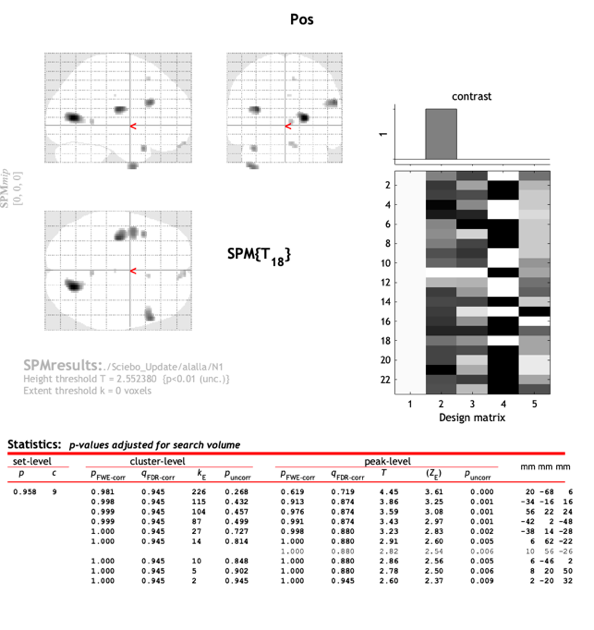 | 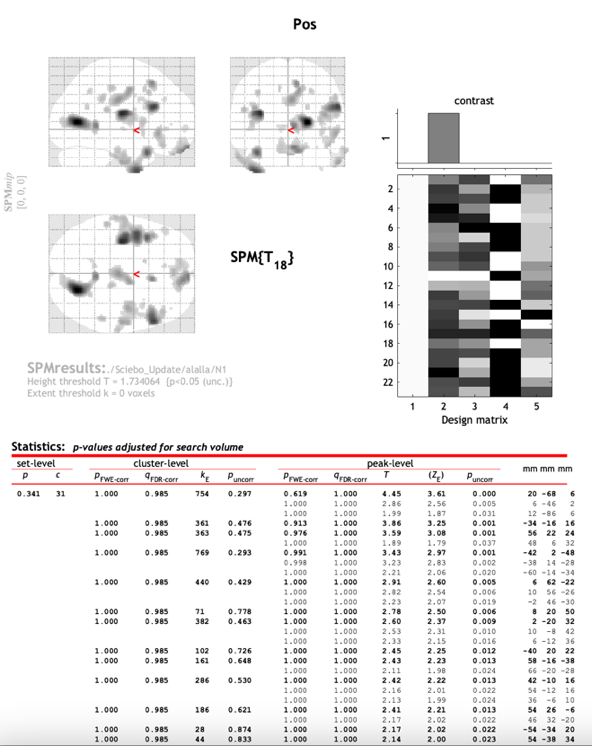 |
| **Positive Effect of N3 in CL10 group** | | | |
| P < .001  T= 3.61 | P < .005  T= 2.87 | P < .01  T= 2.55 | P < .05  T=1.73 |
| 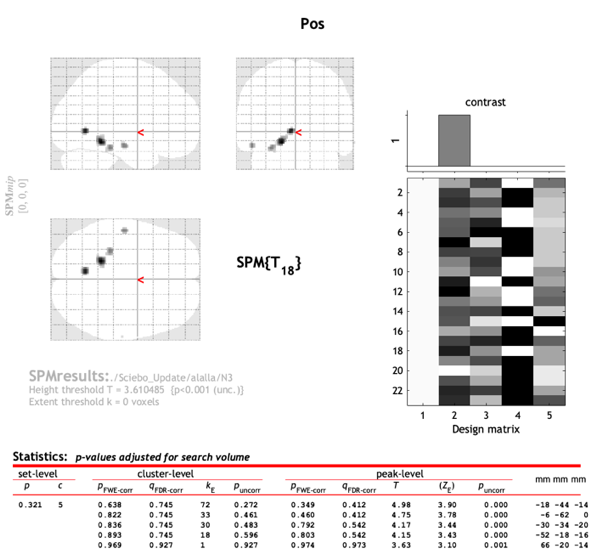 | 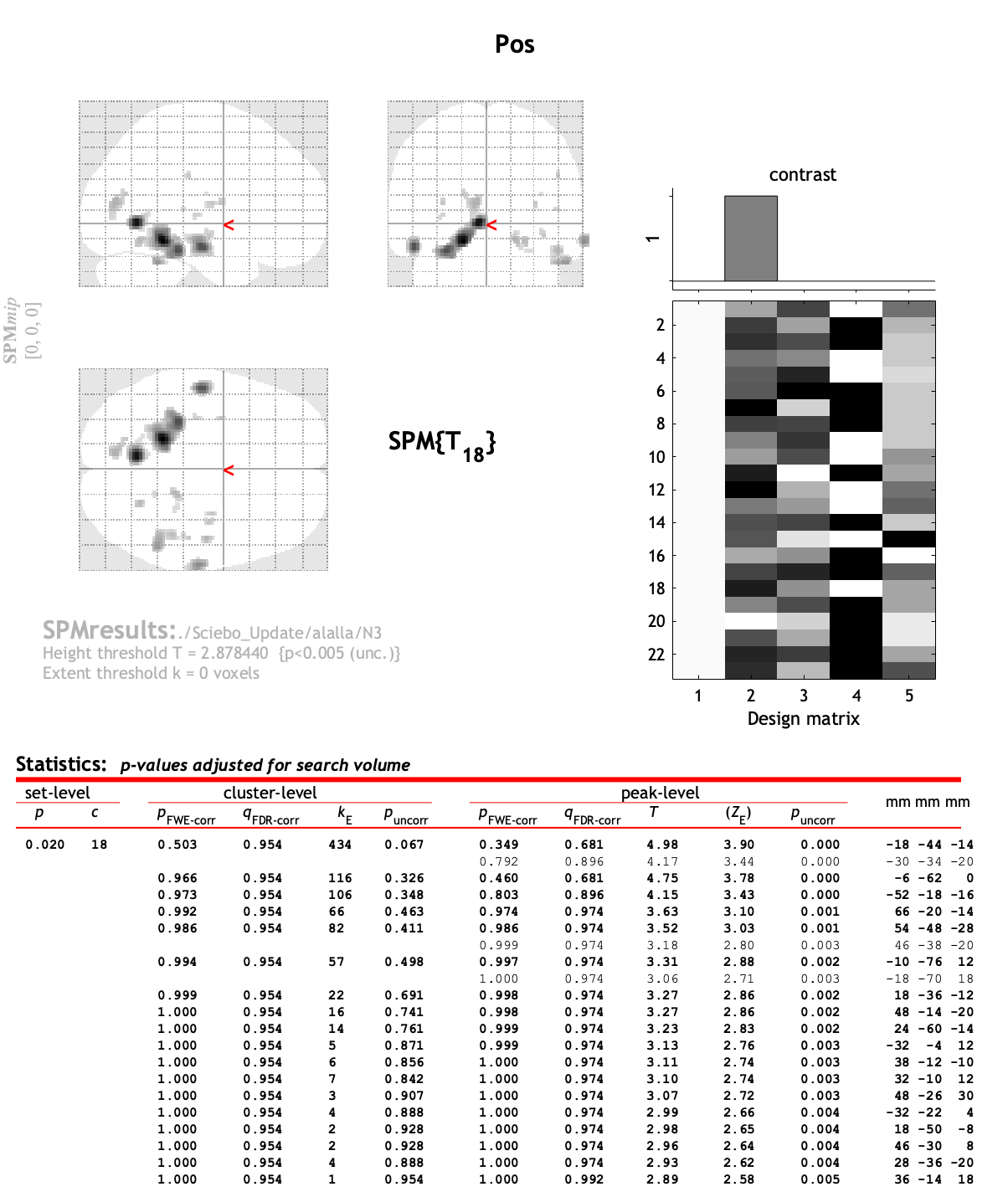 | 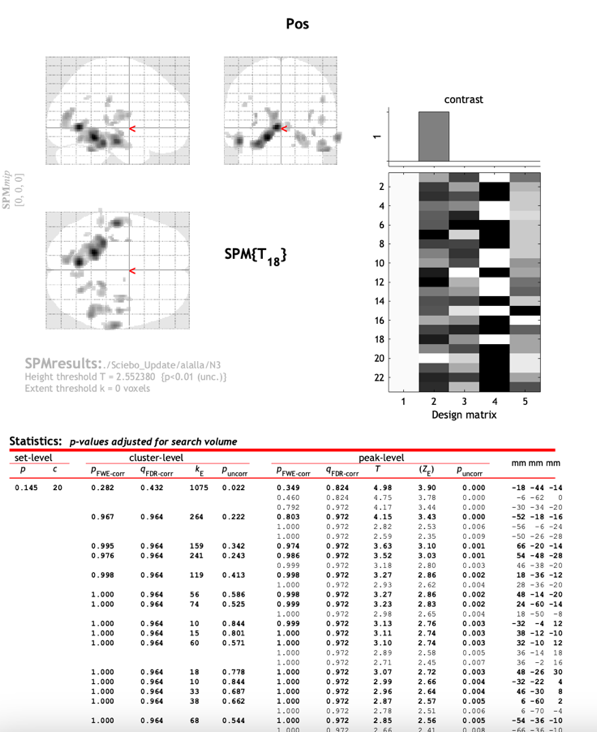 | 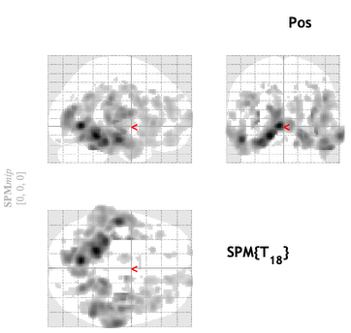 |

**Voxel-wise associations between N1 and N3 duration and z-score maps:**

Z-score maps for each amyloid-positive subject were computed based on the standard deviation and mean voxel maps of the amyloid-negative reference group (CL < 10) using an in-house MATLAB script.

The results of the voxel-wise analyses based on the z-maps yielded similar results as the SUVR maps. Results are presented for the positive contrast of either N1 or N3 at an uncorrected p-threshold of p<.005.

|  | SUVR | Z-Score |
| --- | --- | --- |
| N1 Effect | 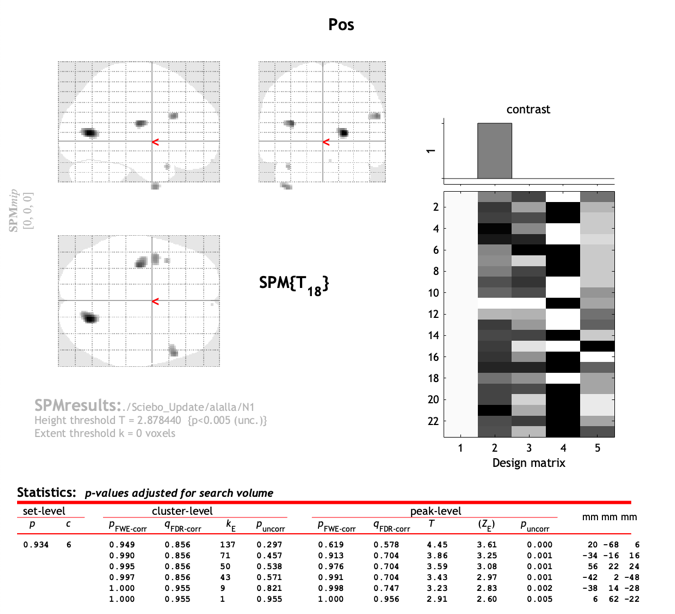 | 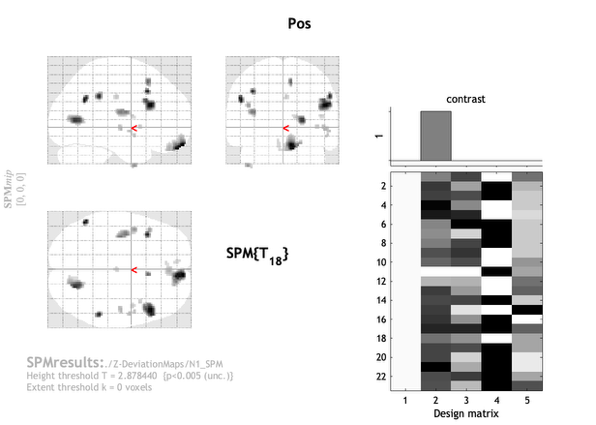 |
| N3 Effect | 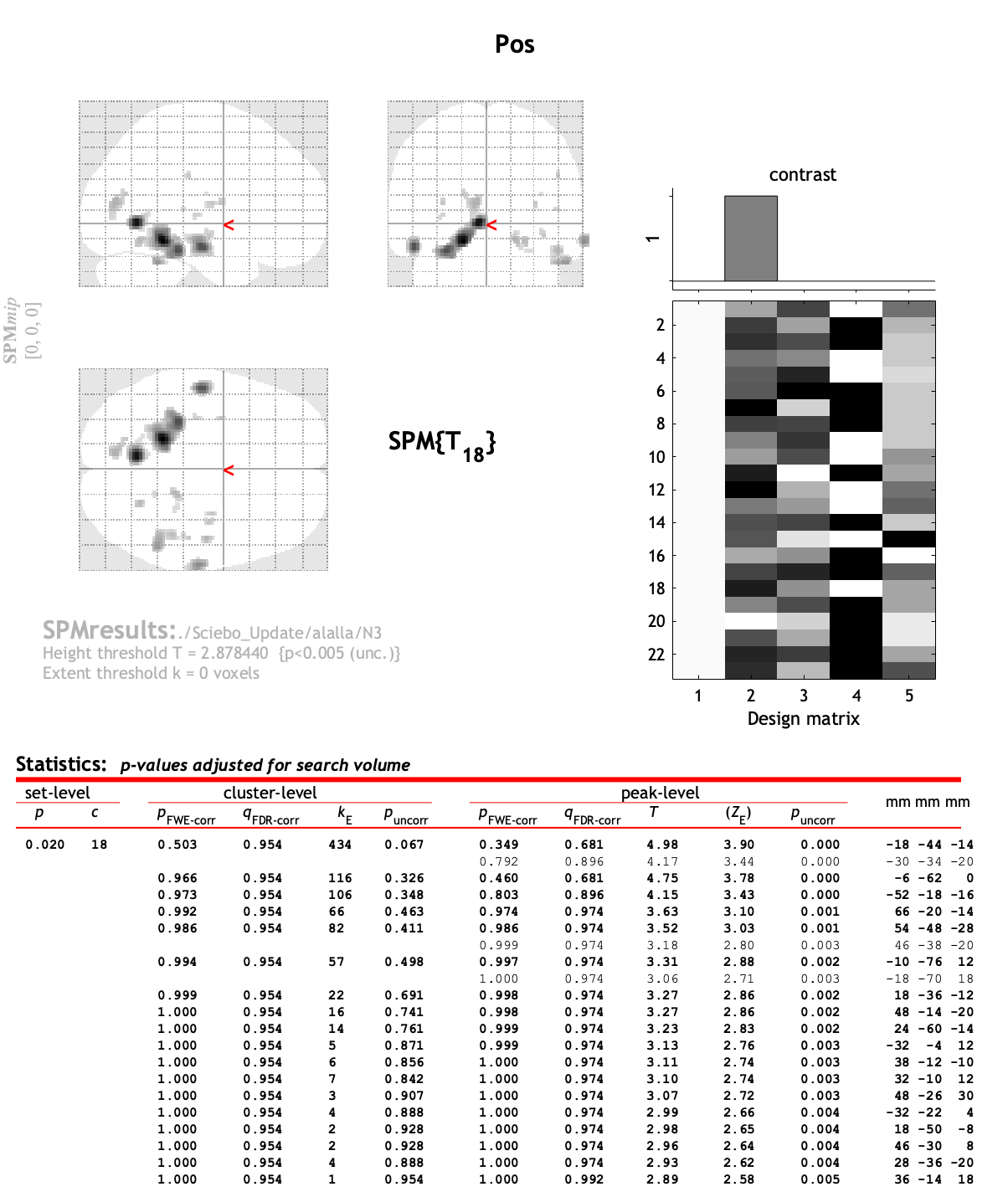 | 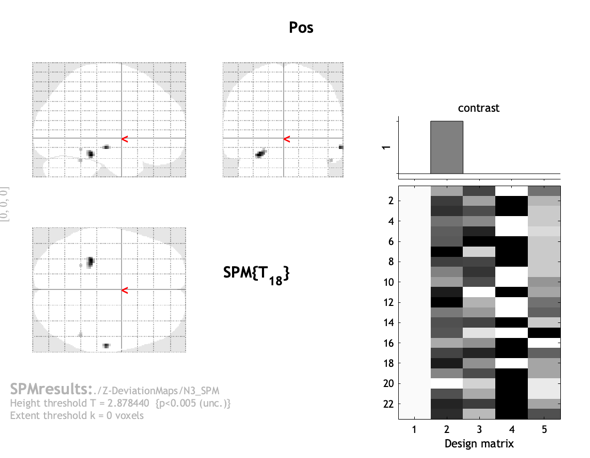 |
